# Comparison of Results of Cycles Treated with Modified Mild Protocol and Short Protocol for Ovarian Stimulation

**DOI:** 10.1155/2014/367474

**Published:** 2014-08-06

**Authors:** F. Coelho, L. F. Aguiar, G. S. P. Cunha, N. Cardinot, E. Lucena

**Affiliations:** ^1^Centro de Infertilidade e Medicina Fetal do Norte Fluminense, Hospital Escola Álvaro Alvim, Rua Barão da Lagoa Dourada, 409-2 ° pavimento, Centro, 28035-210 Campos dos Goytacazes, RJ, Brazil; ^2^Centro Colombiano de Fertilidad y Esterilidad (CECOLFES) S.A.S., Calle 102 No. 14A-15, 56769 Bogota, Colombia

## Abstract

The ovarian stimulation has been applied in order to increase the number of oocytes to compensate for the poor results of in vitro fertilization, allowing the selection of one or more embryos to be transferred. Our aim is to compare the results obtained in IVF/ICSI cycles using the short protocol for controlled ovarian stimulation to the results from the modified mild protocol used in our department. A total of 240 cycles were conducted from January 2010 to December 2011. When comparing both protocols, it could be observed that there was a significant difference in the quantity of gonadotropins doses in the mild protocol and in the short protocol. No significant difference was observed regarding pregnancy rates per cycle, 22% and 26.2%, in short and mild protocols, respectively. The protocols of controlled ovarian stimulation are often associated with high risk of complications such as ovarian hyperstimulation syndrome, excessive emotional stress, high rates of treatment dropouts, and abdominal discomfort. With the data obtained in this study, one can conclude that there are less risks and complications for the patient when using the mild stimulation protocol. It was also observed that in this group there was a slightly higher rate.

## 1. Introduction

Ovarian stimulation is a fundamental part of the technologies involving assisted reproduction. For over 30 years, ovarian stimulation has been applied in order to increase the number of oocytes to compensate for the inefficiency of the in vitro fertilization procedure (IVF), allowing the selection of one or more embryos to be transferred [1–3]. For many years the concept of number generosity of ovules was associated with the also generous number of embryos and directly related to the reproductive treatments prognosis.

In parallel, the number of eggs recruited and the consequent number of resulting embryos contributed to increase the number of multiple pregnancies and the incidence of ovarian hyperstimulation syndrome (OHSS). However, the pregnancy rate did not rise as expected [4].

Likewise, previous studies indicate that the successful embryo implantation depends on an optimal communication between good quality embryos and a receptive endometrium [5]. In IVF the main reason for these poor results of implantation may be the endometrium quality which is affected during the pharmacological treatment (ovarian stimulation and hormone replacement) that is evidenced when comparing both implantation and pregnancy rates among natural cycles and IVF [6].

Recently, a stimulation protocol called mild stimulation protocol has been proposed in order to make the treatment the closest to natural as possible, using low doses of gonadotropins to obtain the success rate similar to the “conventional” stimulus and with fewer side effects to the patients.

This study aims to compare the results obtained in IVF/ICSI cycles using the “short” controlled ovarian stimulation protocol with the results of mild modified protocol used in our department.

## 2. Material and Method

This study was conducted at the Department of Human Reproduction of the Álvaro Alvim Teaching Hospital, in Campos dos Goytacazes, Rio de Janeiro. A total of 160 cycles using the “short” controlled ovarian stimulation protocol and 80 cycles using the mild modified protocol were conducted from January 2010 to December 2011.

In the 80 cycles in which the mild modified protocol for ovarian stimulation was used, 2 tablets of 50 mg clomiphene citrate (Clomid or Indux) were administered daily during a 5-day period associated with urinary gonadotropin 75 IU (Menopur) for 6 days and the pituitary blocking was performed using indomethacin (50 mg Inducid) three times a day for 5 days when a 15-centimeter ovarian follicle was formed. For follicles luteinization a 0.02 mL dose of 0.1 mg leuprolide acetate (Lupron) was administered taking advantage of the endogenous LH when a 17-centimeter or larger ovarian follicle was formed.

From the puncture day on, 3 tablets of 200 mg progesterone (Utrogestan) were administered in a daily basis during a 14-day period, in order to support the luteal phase and provide initial support in case of pregnancy.

In the 160 cycles in which the “short” protocol for ovarian stimulation was used, 0.02-milliliter doses of 0.1 mg leuprolide acetate (Lupron) were administered from the first or second day of the menstrual cycle on, and the ovarian stimulation was initiated on the third day of the cycle using 75 IU/day of recombinant gonadotropins 450 IU/mL (Gonal-F) for 9 days. In addition, 1 daily ampoule of 75 IU urinary gonadotropin (Menopur) was administered during a 10-day period. One ampoule of 250 mg recombinant (r-hCG) chorionic gonadotropin alpha hCG (Ovidrel) was also used. The use of 2 mg estradiol valerate (Primogyna) was initiated when two 17-centimeter ovarian follicles were formed, then it was carried on making use of 2 tablets daily for 16 days until the pregnancy test.

In both groups the needle puncture-aspiration was performed 36 hours after the leuprolide acetate administration, using a 17G COOK needle under a 120 mmHg aspiration pressure, guided by a 7.5 MHz (ALOKA 500-JAPAN) transvaginal ultrasound probe, while the patient was sedated with 10 mg/mL 1% Propofol (Fresofol).

For most patients, the embryo transfer was performed on day 3; however, 12 patients had embryo transfer on day 2 and 10 other patients had embryo transfer on day 5. The embryo quality was monitored daily until the transfer day.

The transfer was performed using SURE-PRO ULTRATM WALLACE transfer catheter coupled to a 1 mL TERUMO syringe and a disposable speculum. The number of preembryos transferred to the uterine cavity followed the Brazilian Resolution of the Federal Council of Medicine (CFM 1957/10) concerning the number of embryos to be transferred where the following determinations are made: (a) up to two embryos for women up to 35 years old; (b) up to three embryos for women between 36 and 39 years old; (c) up to four embryos for 40-year-old women and older.

The statistical analysis consisted of the means and standard errors of the variables for each treatment (short protocol and mild protocol) presentation, as well as the comparison of the means through the *t*-test and of the frequencies through the chi-square test. A 5% significance level was adopted being the analysis performed in the application Statistical and Genetic Analyzes Systems (SAEG, version 9.1).

## 3. Results

**Figure 1:**
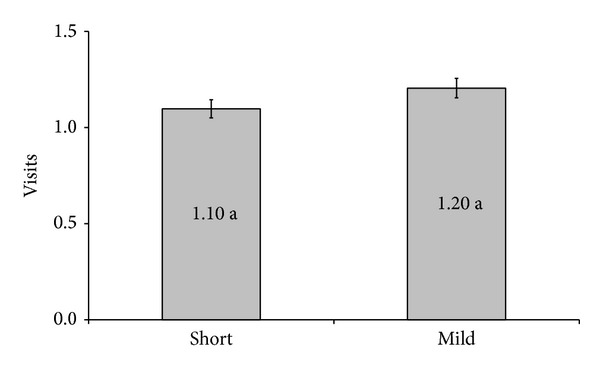
Number of visits to service for monitoring the follicular growth. Means followed by the same letter do not differ significantly through the *t*-test at a 5% probability.

**Figure 2:**
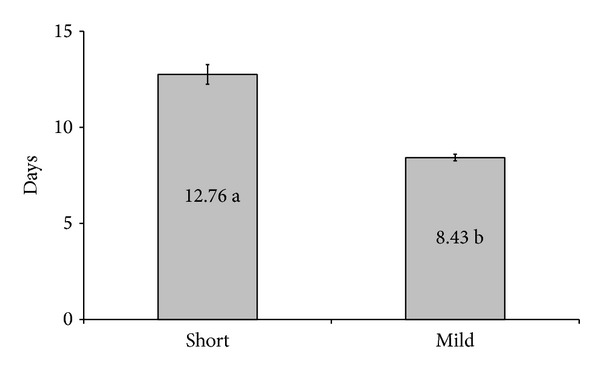
Number of days of stimulation. Means followed by different letters differ significantly through the *t*-test at a 5% probability.

**Figure 3:**
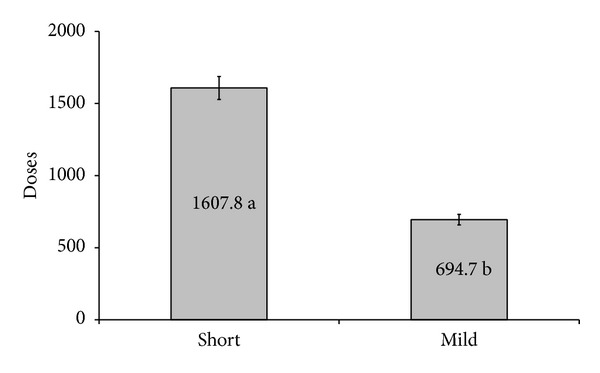
Doses of gonadotropins. Means followed by different letters differ significantly through the *t*-test at a 5% probability.

During the 23-month study period, a total of 240 cycles were performed in our service with patients with a mean age of 34.5. The cycles were divided into two groups: one containing 160 patients stimulated with the “short” protocol for ovarian stimulation and the other one containing 80 patients stimulated with the mild protocol. There was no significant difference between the two groups regarding the number of visits to the service for monitoring the follicular growth (Figure 1). The number of days of stimulation (Figure 2) and the number of gonadotropins doses used (Figure 3) were both significantly lower in the group of patients stimulated with the mild protocol.

**Figure 4:**
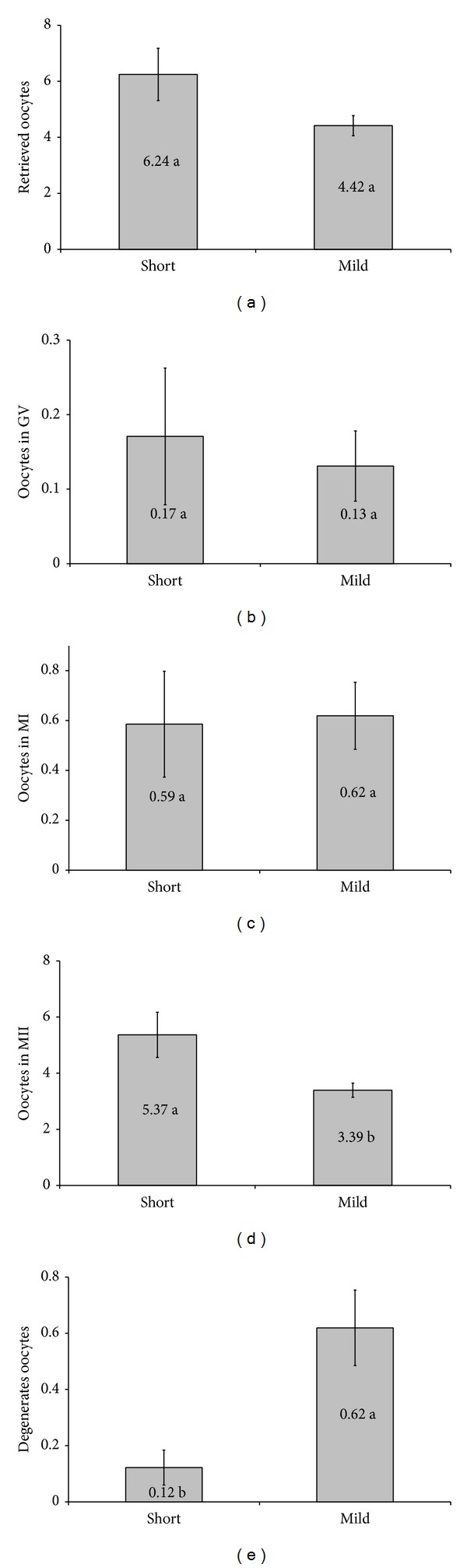
Average number of oocytes per cycle: (a) average of oocytes retrieved, (b) stage of oocytes retrieved at the germinal vesicle (GV), (c) retrieved oocytes in metaphase I (MI), (d) in metaphase II oocytes retrieved (MII), and (e) degenerates oocytes. Means followed by the same letter do not differ significantly by *t*-test at 5% probability.

**Figure 5:**
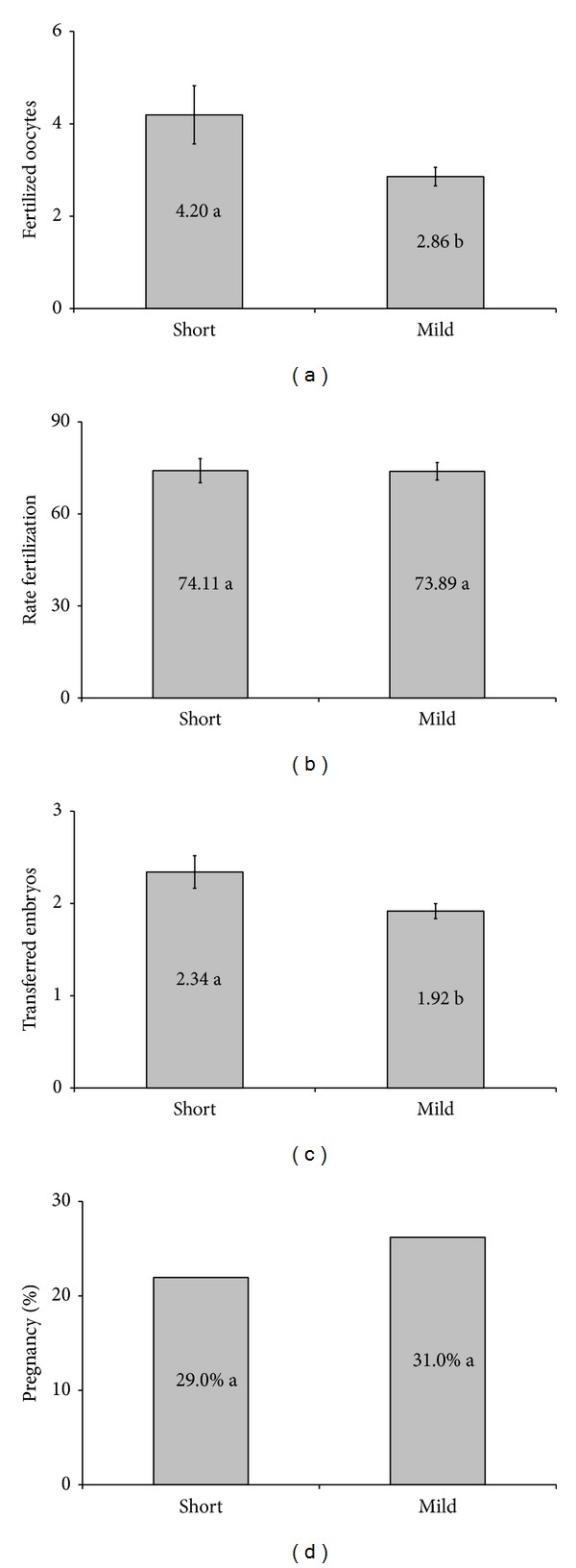
Fertilization rates and pregnancy: (a) average oocytes fertilized, (b) fertilization rate, (c) average number of embryos transferred, and (d) the pregnancy rate. Means followed by the same letter do not differ significantly by *t*-test at 5% probability.

The number of retrieved oocytes (6.24 versus 4.42) was statistically similar in both groups. Analyzing the retrieved oocytes (Figure 4(a)) no significant difference was found in the number of retrieved oocytes in germinal vesicle (GV) (Figure 4(b)) and in immature oocytes (MI) (Figure 4(c)). A significant difference was observed in the quantity of mature oocytes (MII) (5.37 versus 3.39) (Figure 4(d)) and in the quantity of degenerated oocytes (0.12 versus 0,62) (Figure 4(e)). No statistical significance was observed regarding the fertilization rate (Figure 5(b)), even though there was a significant difference in the fertilized ovules quantity (Figure 5(a)). A significant difference was observed in the average number of embryos transferred per cycle (2.34 versus 1.92) (Figure 5(c)). However, no significant difference was noticed regarding the pregnancy rate (Figure 5(d)) between the two groups.

## 4. Discussion

Controlled ovarian stimulation is a key step in assisted reproduction. The concern for oocyte maturation is constant since the end of last century [7].

The development of ovarian stimulation protocols used in conjunction with use of the agonist and antagonist and HCG favored the development of ovarian stimulation protocols. However, success rates have lagged the development of drugs and other stimulus protocols [8].

The conventional GnRH agonist stimulation protocol, resulting in pituitary and ovarian desensitization with the GnRH antagonist stimulation allows the endogenous FSH cycle to occur [9]. Therefore, the cyclic follicular recruitment and gonadotropin-dependent follicles recruited in early stages of growth may continue unimpaired [10, 11].

15, 50, 54, observed that the ovarian stimulation and simultaneous high levels of estradiol were shown to have a negative impact on the potential development of the embryo and its implantation.

The FSH and HCG promote recruitment and maturation of ovules that normally deteriorate in physiological cycles resulting in availability of ova of dubious quality.

This variation of oocyte quality directly affects the embryo quality, a matter of great concern today.

Thus, the increased availability of eggs without the parallel rise of the expected results motivated the transfer of a high number of embryos to compensate for the low success rate. This attempt to offset considerably increased the rate of multiple pregnancy in assisted reproductive technology cycles. This combination, in addition to observed outcomes, resulted in considerable increase in the costs of assisted reproduction treatments and expenditures for perinatal care in such a way that the past 3-4 decades single embryo transfer is a reality and a possibility for mild stimulation.

The purpose of this study was to evaluate the results obtained in IVF/ICSI cycles using the “short” controlled ovarian stimulation protocol with the results of mild modified protocol. The mild modified stimulation protocol we use in our service is a simple and more physiological protocol, which takes advantage of the physiological variations of endogenous gonadotropins, requiring a smaller number of visits for ovarian control and, therefore, reducing any potential risk of medical complications for patients. Ovarian hyperstimulation syndrome (OHSS) corresponds to 1% of complications in assisted reproduction treatments and an important complication of controlled ovarian stimulus [12]. Besides, the SHO controlled ovarian stimulation protocols are often associated with high risk of excessive emotional stress [13] and high rates of treatment dropouts and abdominal discomfort [14]. Moreover, protocols medication used for stimulation is complex, expensive, require weeks of daily injections, and frequent [12].

The mild protocol did not show any complications related to severe ovarian stimulation besides the complaints already related to it as discomfort and abdominal pain. Likewise, there was a reduction in the number of medical visits for ovarian control and consequently, a significant reduction in the cost of treatments. As a result of these observed benefits, the use of LLINs protocols contributed to greater adhesion of patients to treatment proposals. Furthermore, it contributed to the reduction of cases of multiple pregnancies and the need for freezing of surplus embryos; thereby our observations agree with the observations from [5].

Conventional ovarian stimulation may affect mechanisms involved in maintaining accurate chromosome segregation and this is associated with increased incidence of morphological and chromosomal abnormalities [8, 15–18].

Moreover, the mild protocol also relates the best quality embryos and the lowest rate of embryonic aneuploidy when compared to conventional stimulation [19].

Indomethacin can play an important role to overcome a major obstacle in IVF cycles. Furthermore, the safety profile and low cost of this medication make their use attractive.

Indomethacin, an anti-inflammatory nonsteroidal drug, when added to the treatment protocol may prevent ovulation [20]. It inhibits prostaglandin production, especially through the inhibition of cyclooxygenase, the enzyme that catalyzes prostaglandin synthesis and is an essential mediator of ovulation and implantation and also essential to the rupture of the follicle and ovulation. It has been demonstrated that indomethacin administered before ovulation prevents rupture of the follicle, with no apparent lasting effects on the menstrual cycle or FSH, LH, estradiol, and progesterone [13, 21] concern that justified the pituitary suppression with agonist and antagonist [8].

The work in [21] showed that indomethacin administered at the time of positive urinary LH might retard follicular rupture, with an associated reduction in blood flow intrafollicular but without apparent effect on hormone or menstrual status. The mechanism of action of indomethacin, therefore, is probably inhibition of “inflammation” associated with follicular rupture.

The work in [22] contends that indomethacin did not interfere with the effectiveness of assisted reproduction cycles and does not interfere with canceled cycles and, therefore, improves the effectiveness of IVF cycles. The work in [22] also stated that the rate of oocyte retrieval and transfer by procedure were not significantly affected by the use of indomethacin demonstrating that it has no deleterious effects to the embryonic development, and the clinical pregnancy rate per cycle was increased.

In our study we observed that with the use of mild protocol there was a reduction in the number of oocytes retrieved and a greater synchronization of follicles reducing the number of immature oocytes, the number of preembryos transferred and cryopreserved without reducing the overall rates of success treatment IVF confirming the findings of [3]. The observed relationship between low numbers of oocytes and the possibility of success in assisted reproduction cycles after mild stimulation suggests that when some oocytes are obtained, they are likely to represent a homogeneous group of good quality oocytes. This could be the result of the interference with the subtle natural selection of good quality oocytes or minimized exposure of growing follicles to the potentially negative effects of ovarian stimulation.

However, a possible reduction in pregnancy rate was described. This reduction can be confirmed by less comorbidity of mild Protocol for greater comfort, increased safety, reduced likelihood of ovarian hyperstimulation associated with significant reduction of treatment costs and promoting a high rate of adherence to treatment, and the possibility of execution of successive cycles with reduction or pregnancy rate stability, without compromising the rate of drinking at home [2, 18, 23–30].

However, increasing the effectiveness of procedures for IVF and the global trend to limit the number of embryos transferred reduced the need for large amounts of oocytes. The observed relationship between low numbers of oocytes and a high chance of achieving an ongoing pregnancy after mild stimulation suggests that when some oocytes are obtained, they are likely to represent a homogeneous group of good quality oocytes.

When ovarian mild stimulation is combined with a policy of single embryo transfer, the costs associated with pregnancy complications were reduced [29]. The results of this study showed that the use of LLINs modified protocol does not compromise fertilization rates and pregnancy rates if compared to the use of the “short” stimulation protocol. However, the number of days of stimulation, the financial cost, the complications, and the number of doses of gonadotropins used were significantly shorter.

A decrease in the pregnancy rate in patients with a more pronounced ovarian response to stimulation using mild may be associated with the occurrence of premature LH rises [1]. The occurrence of premature LH elevation has a negative impact on the possibility of achieving an ongoing pregnancy [16].

The trigger ovulation, hCG, which is thought to have an effect of “proestablishment” is becoming a prime target in the search for substances with a negative role in endometrial receptivity [10, 31].

Studies that have examined the fate of oocytes demonstrated that prolonged exposure to hCG in the proliferative phase adversely affects the pregnancy rate [32].

Likewise, the “natural” ovulation determined by monitoring the LH surge results in higher implantation rates and ongoing pregnancy in normoovulatory women versus those in which ovulation is triggered with hCG IUI and frozen embryo transfers [33].

The implantation is a highly complex process that requires close synchronization between the development of the embryo and the endometrium [34].

Moreover, the endometrium could respond to embryonic signals such as hCG, to facilitate preparation for implantation.

The supporting evidence regarding a possible adverse effect of supraphysiological levels of steroids in endometrial receptivity [4, 9], corpus luteum function [14, 35], oocyte, and embryo quality [36] indicates that the response of limited ovarian stimulation may have a beneficial effect on the potential of implantation.

In assisted reproduction treatment one of the main reasons for the poor results in the implantation of the endometrium is the quality that is affected during pharmacological treatment (ovarian stimulation and hormone replacement) that is emphasized when comparing the rates of implantation and pregnancy rates between natural cycles and FIV [37]. According to [34, 38] increased progesterone during the late follicular phase has been considered a negative factor for clinical outcomes. The mechanism that may be attached to this observation can be the fact that high serum levels of progesterone on the day of hCG administration induce both the advanced endometrial maturation and the differential expression of endometrium genes, which may be related to the deployment failure [39–41].

The work in [9] noted that considering the concentrations of estradiol isolated was independent to the number of oocytes retrieved. Implantation rates and pregnancy were significantly reduced when estradiol concentrations were elevated.

In our reality, a developing country, the main barrier to access for couples to acquire assisted reproduction treatments, is the financial cost involved, because there is no coverage for public assistance. Thus, only a small privileged fraction of the population has access to these treatments.

Thus the mild protocol we use in our service associated with the blockade of ovulation with indomethacin and its triggering with GnRH agonist substantially reduces the cost of treatment and possible complications expenditures when compared to the SHO. The use of LLINs as trigger associated with using the endogenous LH agonist, as no pituitary suppression, eliminates the risk of OHSS. Besides, the use of low doses of gonadotropin and no use of hCG often associated with the pathophysiology of OHSS. Thus the financial expenses related complications by any hospital admissions are also eliminated. All this makes for easy access for couples who generally would be limited by the financial cost of treatment.

Likewise, when the programs of assisted reproduction are associated with a single embryo transfer, it reduces the rate of multiple pregnancies and subsequent expenses with neonatal intensive treatment systems.

In conclusion, mild protocol did not interfere with treatment outcomes, achieving greater compliance and cost reduction and it acts as an important gateway to assisted reproductive treatments in developing countries and may be an option for models of public assistance to infertility in these countries.

## References

[1] Borm G, Mannaerts B (2000). Treatment with gonadotrophin-releasing hormone antagonist ganirelix in women undergoing ovarian stimulation with recombinant follicle stimulating hormone is effective, safe and convenient: results of a controlled, randomized, multicentre trial. *Human Reproduction*.

[2] Branigan EF, Estes MA (2000). Minimal stimulation IVF using clomiphene citrate and oral contraceptive pill pretreatment for LH suppression. *Fertility and Sterility*.

[3] Fauser BC, Devroey P, Yen SS (1999). Minimal ovarian stimulation for IVF: appraisal of potential benefits and drawbacks. *Human Reproduction*.

[4] Devroey P, Bourgain C, Macklon NS, Fauser BCJM (2004). Reproductive biology and IVF: ovarian stimulation and endometrial receptivity. *Trends in Endocrinology and Metabolism*.

[5] Reindollar RH, Goldman MB (2012). Gonadotropin therapy: a 20th century relic. *Fertility and Sterility*.

[6] Keay SD, Liversedge NH, Mathur RS, Jenkins JM (1997). Assisted conception following poor ovarian response to gonadotrophin stimulation. *British Journal of Obstetrics and Gynaecology*.

[7] Gemzell CA, Roos P, Loeffler FE, Behrman SJ, Kistner RW (1975). Follicle stimulating hormone extracted from human pituitary. *Progress in Infertility*.

[8] Roberts R, Iatropoulou A, Ciantar D (2005). Follicle-stimulating hormone affects metaphase I chromosome alignment and increases aneuploidy in mouse oocytes matured in vitro. *Biology of Reproduction*.

[9] Simon C, Cano F, Valbuena D, Remohi J, Pellicer A (1995). Clinical evidence for a detrimental effect on uterine receptivity of high serum oestradiol concentrations in high and normal responder patients. *Human Reproduction*.

[10] Fauser BCJM, Van Heusden AM (1997). Manipulation of human ovarian function: Physiological concepts and clinical consequences. *Endocrine Reviews*.

[11] McGee EA, Hsueh AJW (2000). Initial and cyclic recruitment of ovarian follicles. *Endocrine Reviews*.

[12] Pelinck MJ, Vogel NEA, Hoek A (2006). Cumulative pregnancy rates after three cycles of minimal stimulation IVF and results according to subfertility diagnosis: a multicentre cohort study. *Human Reproduction*.

[13] Hester KE, Harper MJK, Duffy DM (2010). Oral administration of the cyclooxygenase-2 (COX-2) inhibitor meloxicam blocks ovulation in non-human primates when administered to simulate emergency contraception. *Human Reproduction*.

[14] Fauser BCJM, Devroey P (2003). Reproductive biology and IVF: ovarian stimulation and luteal phase consequences. *Trends in Endocrinology and Metabolism*.

[15] Eppig JJ, O'Brien MJ, Pendola FL, Watanabe S (1998). Factors affecting the developmental competence of mouse oocytes grown in vitro: follicle-stimulating hormone and insulin. *Biology of Reproduction*.

[16] Humaidan P, Bungum L, Bungum M, Andersen CY (2002). Ovarian response and pregnancy outcome related to mid-follicular LH levels in women undergoing assisted reproduction with GnRH agonist down-regulation and recombinant FSH stimulation. *Human Reproduction*.

[17] Munné S, Magli C, Adler A (1997). Treatment-related chromosome abnormalities in human embryos. *Human Reproduction*.

[18] Van Blerkom J, Davis P (2001). Differential effects of repeated ovarian stimulation on cytoplasmic and spindle organization in metaphase II mouse oocytes matured *in vivo* and *in vitro*. *Human Reproduction*.

[19] Haaf T, Hahn A, Lambrecht A (2009). A high oocyte yield for intracytoplasmic sperm injection treatment is associated with an increased chromosome error rate. *Fertility and Sterility*.

[20] Rijken-Zijlstra TM, Haadsma ML, Hammer C (2013). Effectiveness of indometacin to prevent ovulation in modified natural-cycle IVF: a randomized controlled trial. *Reproductive BioMedicine Online*.

[21] Athanasiou S, Bourne TH, Khalid A (1996). Effects of indomethacin on follicular structure, vascularity, and function over the periovulatory period in women. *Fertil. Steril*.

[22] Kadoch IJ, Al-Khaduri M, Phillips SJ (2008). Spontaneous ovulation rate before oocyte retrieval in modified natural cycle IVF with and without indomethacin. *Reproductive BioMedicine*.

[23] Baart EB, Martini E, Eijkemans MJ (2007). Milder ovarian stimulation for in-vitro fertilization reduces aneuploidy in the human preimplantation embryo: a randomized controlled trial. *Human Reproduction*.

[24] Diedrich K, Felberbaum R (1998). New approaches to ovarian stimulation. *Human Reproduction*.

[25] Katz-Jaffe MG, Trounson AO, Cram DS (2005). Chromosome 21 mosaic human preimplantation embryos predominantly arise from diploid conceptions. *Fertility and Sterility*.

[26] Nargund G, Frydman R (2007). Towards a more physiological approach to IVF. *Reproductive BioMedicine Online*.

[27] Olivennes F, Frydman R (1998). Friendly IVF: the way of the future?. *Human Reproduction*.

[28] Olivennes F (2002). GnRH antagonists: do they open new pathways to safer treatment in assisted reproductive techniques?. *Reproductive Biomedicine Online*.

[29] Pennings G, Ombelet W (2007). Coming soon to your clinic: patient-friendly ART. *Human Reproduction*.

[30] Ubaldi FM, Rienzi L, Baroni E (2007). Hopes and facts about mild ovarian stimulation. *Reproductive BioMedicine Online*.

[31] Kyrou D, Kolibianakis EM, Fatemi HM (2012). Spontaneous triggering of ovulation versus HCG administration in patients undergoing IUI: a prospective randomized study. *Reproductive BioMedicine Online*.

[32] Prapas N, Tavaniotou A, Panagiotidis Y (2009). Low-dose human chorionic gonadotropin during the proliferative phase may adversely affect endometrial receptivity in oocyte recipients. *Gynecological Endocrinology*.

[33] Shapiro BS, Daneshmand ST, Garner FC, Aguirre M, Hudson C, Thomas S (2011). Evidence of impaired endometrial receptivity after ovarian stimulation for in vitro fertilization: a prospective randomized trial comparing fresh and frozen-thawed embryo transfer in normal responders. *Fertility and Sterility*.

[34] Bermejo A, Iglesias C, Ruiz-Alonso M (2014). The impact of using the combined oral contraceptive pill for cycle scheduling on gene expression related to endometrial receptivity. *Human Reproduction*.

[35] Beckers NGM, Platteau P, Eijkemans MJ (2006). The early luteal phase administration of estrogen and progesterone does not induce premature luteolysis in normo-ovulatory women. *European Journal of Endocrinology*.

[36] Valbuena D, Martin J, De Pablo JL, Remohí J, Pellicer A, Simón C (2001). Increasing levels of estradiol are deleterious to embryonic implantation because they directly affect the embryo. *Fertility and Sterility*.

[37] Paulson RJ, Sauer MV, Lobo RA (1990). Embryo implantation after human in vitro fertilization: importance of endometrial receptivity. *Fertility and Sterility*.

[38] Papanikolaou EG, Pados G, Grimbizis G (2012). GnRH-agonist versus GnRH-antagonist IVF cycles: Is the reproductive outcome affected by the incidence of progesterone elevation on the day of HCG triggering? A randomized prospective study. *Human Reproduction*.

[39] Elnashar AM (2010). Progesterone rise on the day of HCG administration (premature luteinization) in IVF: an overdue update. *Journal of Assisted Reproduction and Genetics*.

[40] Schoolcraft W, Sinton E, Schlenker T, Huynh D, Hamilton F, Meldrum DR (1991). Lower pregnancy rate with premature luteinization during pituitary suppression with leuprolide acetate. *Fertility and Sterility*.

[41] Silverberg KM, Burns WN, Olive DL, Riehl RM, Schenken RS (1991). Serum progesterone levels predict success of in vitro fertilization/embryo transfer in patients stimulated with leuprolide acetate and human menopausal gonadotropins. *Journal of Clinical Endocrinology and Metabolism*.

